# Implementation of Web-Based Respondent-Driven Sampling among Men Who Have Sex with Men in Vietnam

**DOI:** 10.1371/journal.pone.0049417

**Published:** 2012-11-12

**Authors:** Linus Bengtsson, Xin Lu, Quoc Cuong Nguyen, Martin Camitz, Nguyen Le Hoang, Tuan Anh Nguyen, Fredrik Liljeros, Anna Thorson

**Affiliations:** 1 Department of Public Health Sciences, Karolinska Institutet, Stockholm, Sweden; 2 Department of Sociology, Stockholm University, Stockholm, Sweden; 3 Family Heath International, Hanoi, Vietnam; 4 iSEE, Hanoi, Vietnam; 5 Hanoi Medical University, Hanoi, Vietnam; Tulane University, United States of America

## Abstract

**Objective:**

Lack of representative data about hidden groups, like men who have sex with men (MSM), hinders an evidence-based response to the HIV epidemics. Respondent-driven sampling (RDS) was developed to overcome sampling challenges in studies of populations like MSM for which sampling frames are absent. Internet-based RDS (webRDS) can potentially circumvent limitations of the original RDS method. We aimed to implement and evaluate webRDS among a hidden population.

**Methods and Design:**

This cross-sectional study took place 18 February to 12 April, 2011 among MSM in Vietnam. Inclusion criteria were men, aged 18 and above, who had ever had sex with another man and were living in Vietnam. Participants were invited by an MSM friend, logged in, and answered a survey. Participants could recruit up to four MSM friends. We evaluated the system by its success in generating sustained recruitment and the degree to which the sample compositions stabilized with increasing sample size.

**Results:**

Twenty starting participants generated 676 participants over 24 recruitment waves. Analyses did not show evidence of bias due to ineligible participation. Estimated mean age was 22 years and 82% came from the two large metropolitan areas. 32 out of 63 provinces were represented. The median number of sexual partners during the last six months was two. The sample composition stabilized well for 16 out of 17 variables.

**Conclusion:**

Results indicate that webRDS could be implemented at a low cost among Internet-using MSM in Vietnam. WebRDS may be a promising method for sampling of Internet-using MSM and other hidden groups.

## Introduction

Men who have sex with men (MSM) has emerged as a key population in the global HIV epidemic [1], [2], [3]. Modeling work on the Asian epidemic points to a scenario in which 42 percent of all new HIV infections in Asia will occur among MSM by 2020 [4]. While population-based surveys in countries with generalized epidemics have generated vast amounts of data on sexual behavior [5], studies on MSM and other hidden populations struggle to generate representative samples and adequate sample sizes [1]. The lack of representative data of MSM risk-behavior severely hinders an understanding of the underlying dynamics of the MSM epidemics and prevents an evidence-based response. New methods for representative sampling of MSM and other hidden groups are thus needed.

Respondent-driven sampling (RDS) was developed to overcome sampling challenges in studies of populations for which a sampling frame is difficult or impossible to define, such as MSM, injecting drug users (IDU), and sex workers (SW) [6], [7], [8], [9], [10], [11]. An RDS study starts by purposively selecting a handful of participants who are known members of the study population. These “seeds” are given invitation coupons (usually three) to distribute to other members of the population. These members are in turn given three new coupons to distribute. Monetary incentives are usually given both for participation and recruitment.

RDS resembles “snowball sampling” [12] but differs from it in several important respects. The RDS incentive system and the limited number of invitation coupons per participant allow the creation of long recruitment chains. If the sampling conforms to methodological assumptions, the proportion of the sample with a certain characteristic stabilizes at a level determined by the characteristics of the population, independently of the characteristics of the seeds [7]. Furthermore, snowball sampling systematically oversamples individuals with many contacts. All individual properties correlated with the number of contacts within the group under study will hence be over or under sampled in a snowball sample. In contrast, during an RDS study researchers record an estimate of each person’s social network size and adjust for this bias. Participants are also not required to name or identify their contacts, as is often the case in snowball sampling. Instead participants can pass invitation coupons to any of their contacts at their own discretion. They receive a reward when their contacts participate in the survey, serving to increase participation rates and decrease selection bias. The RDS method has been shown to be analytically unbiased under a limited number of assumptions. Extensive methodological research is ongoing to investigate how well these assumptions are met during real-life implementation, how to best estimate variance and what determines the suitability of the method in the local context [9], [13], [14], [15], [16], [17], [18], [19], [20].

Although RDS in certain contexts has clear advantages over other sampling methods [21], the standard implementation of the method has several limitations, including: 1) individuals with a behavior that is stigmatized, illegal, or associated with high privacy concerns may be unwilling to access survey offices physically and may thus be underrepresented in the sample; 2) persons from middle- and upper income levels may not be sufficiently incentivized by the study rewards, given the time and effort required to participate; 3) the geographic area of study needs to be small enough to allow participants to travel to the study sites; and 4) RDS studies are, like other field survey methods, relatively expensive since they require the presence of trained staff for extended periods of time and need to be repeated at several sites to generate national or regional data,

Sampling participants though the Internet can mitigate some of these disadvantages by allowing people to participate anonymously and with little effort. Online sampling also allows for vast geographic coverage and may be carried out at markedly lower cost than standard field surveys. However, current methods of Internet-based sampling of hidden groups enroll participants through self-selection, which may cause important bias [22]. Usually, a so-called banner add is put on a web page, e.g. a site for gay men. Persons accessing this site then click the banner to volunteer for the study. These surveys can have participation rates as low as a few in a thousand to a few in a hundred out of registered users [23], [24].

Web-based RDS (henceforth webRDS) can potentially circumvent both the disadvantages of standard RDS as well as disadvantages inherent in current Internet-based sampling methods of hidden groups. There are three published webRDS surveys, two involving students at Cornell University [16], [25] and one among users of Facebook in the U.S [26]. The results of these studies showed that RDS estimates agreed relatively well with the true characteristics study population with the exception, in the Facebook survey, of undersampling of participants who self-identified as Hispanic/Latino, African American/Black American and were of lower education levels. These surveys did not, however, target a hidden population. We aimed to implement and evaluate webRDS for sampling and surveying of a hidden and stigmatized population, Internet-using MSM in Vietnam.

## Materials and Methods

### General Study Design

The survey was cross-sectional, performed online and carried out between February 18 and April 12, 2011, applying web-based respondent driven sampling (webRDS).

### Inclusion Criteria and Population Delineation

Eligible participants were adult men (18 years and above) who had ever had any type of sex (including oral sex and mutual masturbation) with another man, had not previously participated in the survey, and were living in Vietnam at the time of the study. The Internet-using part of this group formed the population to which the sample aimed to generalize.

### MSM and Internet Use in Vietnam

Internet access in Vietnam costs approximately 0.15 USD per hour at Internet cafés. MSM in Vietnam are stigmatized [27], and HIV prevalence in the group has been estimated at 14–20% and 14–16% in Hanoi and Ho Chi Minh City, respectively (2009) [28]. Internet use as a proportion of the population in Vietnam was 27% in 2010 (24 million persons) and 60% and 50%, respectively, in the large urban areas of Hanoi and Ho Chi Minh City [29]. Internet use among MSM in general may be considerably higher than in the general population [30]. Ninety-four percent of MSM in an offline RDS in Hanoi stated that they used the Internet [31]. The Internet in Vietnam provides an important environment in which MSM communicate with each other and meet partners.

### Sampling

The study was performed in collaboration with a local research organization in Vietnam working to promote LGBT and ethnic minority rights (iSEE). iSEE has an extensive knowledge and contact network among MSM community groups and a close collaboration with web administrators of Vietnamese LGBT web sites. Fifteen seeds, who were recruited through these networks, initiated the survey and a further five seeds were added two weeks later to increase the speed of recruitment. Six seeds came from Ho Chi Minh City, ten from Hanoi and four from Hoa Binh. Nineteen out of the 20 seeds had attended some kind of education after high school (vocational training, college or university). Participants received, from their recruiter, an invitation message with a login code and a web address. They logged in, accessed detailed information about the study, approved participation and eligibility and answered a written questionnaire. Participants could then compare their own answers to aggregated answers of earlier participants, displayed in informative bar charts. On the last page the participants were encouraged to recruit MSM friends by providing an e-mail or Yahoo Messenger address (popular for communications in Vietnam), and being automatically sent four invitation messages, which could be forwarded to MSM friends. The messages were also displayed on the screen and could be copied for sending by other preferred means. Text both on the web site and in the email/Yahoo! chat messages emphasized that only MSM living in Vietnam and of age 18 years or above were allowed to participate. A warning was included saying that advanced checks were applied and that failure to follow the recruitment rules would mean loss of compensation. No restriction was given as to whether the recruiter knew each other in real life or only through the Internet. Reminders to recruit were sent out two and four days after completing the survey. Participants were informed that they had seven days to recruit and were given rewards for recruitments that took place during that time. Some participants took the survey at a later time point. They were retained in the sample and the persons they recruited were given standard compensation.

### Web Site

The graphic design of the web site aimed at giving a professional and friendly impression without strong MSM connotations.

### Incentives and Recruitment Stimuli Included the Following

1) 2.45 USD (50,000 VND) as credit on the participant’s SIM card and the same amount for each successful recruitment of an MSM friend (maximum four); 2) the option of donating the monetary reward to an MSM community organization chosen by the participant; 3) a lottery with the possibility of winning an iPad; 4) text emphasizing participation in order to support MSM in Vietnam; and 5) being able to compare one’s own answers to those of other participants in simple, informative and anonymous charts. Eight questions were included in the questionnaire specifically to stimulate the participants’ interest in comparing themselves with other participants.

### Piloting and Early Versions of the System

The web site and recruitment system was extensively pilot tested. Interviews and focus-group discussions among MSM were performed to understand social networks among MSM, online interaction and to decide on appropriate incentives. Two versions of the webRDS site were used for sampling before the study described in this paper was carried out. These webRDS systems differed in that they had a less advanced graphic design and smaller incentives. In the first survey in 2009, recruitment died out after a maximum of 5 waves (25 participants, 15 seeds). The second time, recruitment improved but stopped after 5 waves (84 participants, 15 seeds).

### Data Collection

The questionnaire contained 17 questions, including number of sexual partners in the past 6 months, sexual partner preferences (prefer as sexual partners only men, men to women, women to men or only women), the duration of the respondent’s longest relationship, opinion on legalizing same-sex marriage in Vietnam (for or against), frequency of Internet-use, socio-demographic characteristics, network size (see separate heading), relationship between the participant and his recruiter (stranger, acquaintance, friend, close friend, lover/ex-lover, or relative), and the social context in which the participant got to know his recruiter. Logical checks with error messages were used for interdependent questions. Only positive integers were allowed for numeric answers. All questions included a “don’t want to answer” option and all questions needed to be answered. Participants who wanted to receive rewards filled out contact details and a personal identifier (telephone number, email or Yahoo! Messenger address, and the last three digits of their nine-digit ID number). Time points at which each participant loaded the web pages was stored to facilitate identification of ineligible submissions, including unserious attempts to answer the questionnaire or the same person trying to answer more than one questionnaire to receive additional rewards.

### Analyses of Duplicated Submissions, Data Cleaning and Analysis

9.6% of completed surveys (65 surveys) included a stated age below 18 years, or a telephone number, e-mail or Yahoo! Chat address that had previously been registered in the system. We defined these as “invalid”. We excluded seeds (customary in RDS analysis [8]) together with the aforementioned invalid submissions to produce a cleaned sample. From this sample we estimated, in Matlab, population proportions using the current state of the art estimator, RDSII, which requires only information on the sample compositions and the social network sizes of the participants [10]. We have not included confidence intervals in this paper since there is currently no consensus on how to best estimate RDS design effects.

We checked all surveys for other signs of duplication or invalidity by flagging surveys containing a repeated IP number, deviating answers (as described below), or short completion times. We analyzed the sensitivity of the estimates to inclusion and exclusion of these flagged submissions. Specifically we compared the RDS II estimates generated from the full sample of non-seed submissions with valid age with the RDS II estimates generated from groups with progressively stricter inclusion criteria according to the following: 1) exclusion of submissions with a repeated email, Yahoo! Chat ID or telephone number (forming the cleaned sample above); 2) additionally excluding repeated IP numbers; and 3) additionally excluding submissions with short completion times (< three minutes), submissions stating no education (rare in Vietnam), or submission stating six-month partner numbers above 1,000. Differences were small between the groups. Details are included in the supplementary material. For all estimates in the supplementary material the maximum absolute differences when comparing the full sample to the groups with progressively stricter inclusion criteria were 6.6%.

### Personal Network Size

We asked participants for the number of MSM they had interacted with in any way during the past seven days (including on the phone, Internet, or in person). We then asked how many of these persons they believed used the Internet. We chose the seven-day timeframe to reflect the potential high frequency of contacts online. We used the second network question to define the participants’ personal network. We replaced missing personal network size data with the RDSII-estimated average network size from submissions with non-missing network data. The average network size was 5.5 persons.

### Evaluation and Analyzes of Equilibrium

As there is no gold standard by which to validate the sampling, we evaluated the system in terms of its success in generating sustained recruitment, the degree to which the sample compositions stabilized with increasing sample size (independence of the sample from the seeds), and finally, in the discussion, we contrast the sample compositions with results from other surveys.

We analyzed whether equilibrium was achieved in two ways. We first used the standard criterion from the RDS study literature [32]. This criterion requires the sampling process to have reached a certain number of waves. The number of waves required is, for each variable, determined by the number of steps required by a first-order Markov process to reach a less than a two percent relative difference between its value at a given step and its value after an infinite number of steps. The transition probabilities used to calculate the values of the Markov process are the averaged transition probabilities in the study’s recruitment chains. Second, we produced plots of the changes in the sample compositions as sample size increased.

### Ethics

All information about the survey was available on all web pages except the log-in page and could be accessed at any time. All pages included a log-out button, which automatically removed traces of the survey from the computer and transported the user to a search engine. Browser history has to be deleted manually by the user and participants were given detailed instructions on how to do so. Telephone and chat support were available. IP addresses were converted to a unique anonymous code using a one-way encryption algorithm, and the original IP numbers were deleted. Login passwords were only valid for a single session and could not be used on two computers simultaneously. Communication between the users and the server was encrypted. Graphs with aggregated survey results were updated with data from a large number of participants at a time and displayed so that it was impossible to understand what others answered. The study was approved by the Hanoi Medical University Review Board for Bio-Medical Research.

## Results

### Recruitment Dynamics

**Figure 1:**
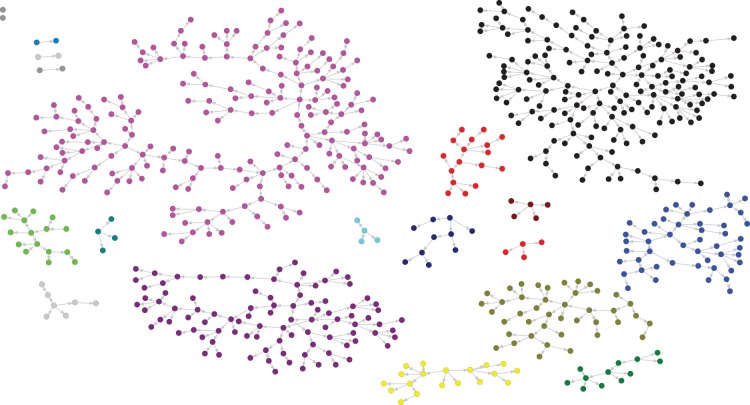
The recruitlment chains of submitted surveys. Each color represents a separate recruitment chain. Two seeds did not generate further participants.

676 study participants submitted a survey during the study period. The length of recruitment chains varied from 1 to 24 waves (excluding seed wave). Eight recruitment chains (out of 20) reached more than five waves (Fig. 1).

**Figure 2:**
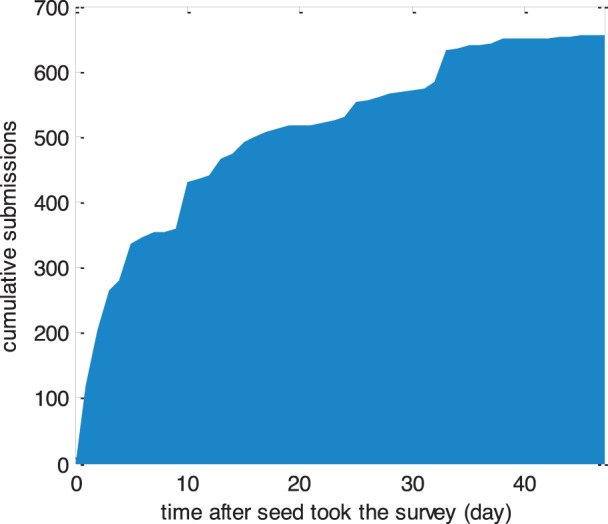
Cumulative number of survey submissions over time.

Five seeds were added 14 days after the first group (see methods). For clarity of presentation we backdated the start date of these five seeds 14 days so that all seeds could be considered to have started on the same day. Using this adjustment, the site received slightly less than 500 submissions during the first two weeks of sampling. The daily number of submissions then gradually decreased and about 100 surveys were submitted during the last 20 days, after which submissions stopped by itself (Fig. 2).

### Equilibrium

**Figure 3:**
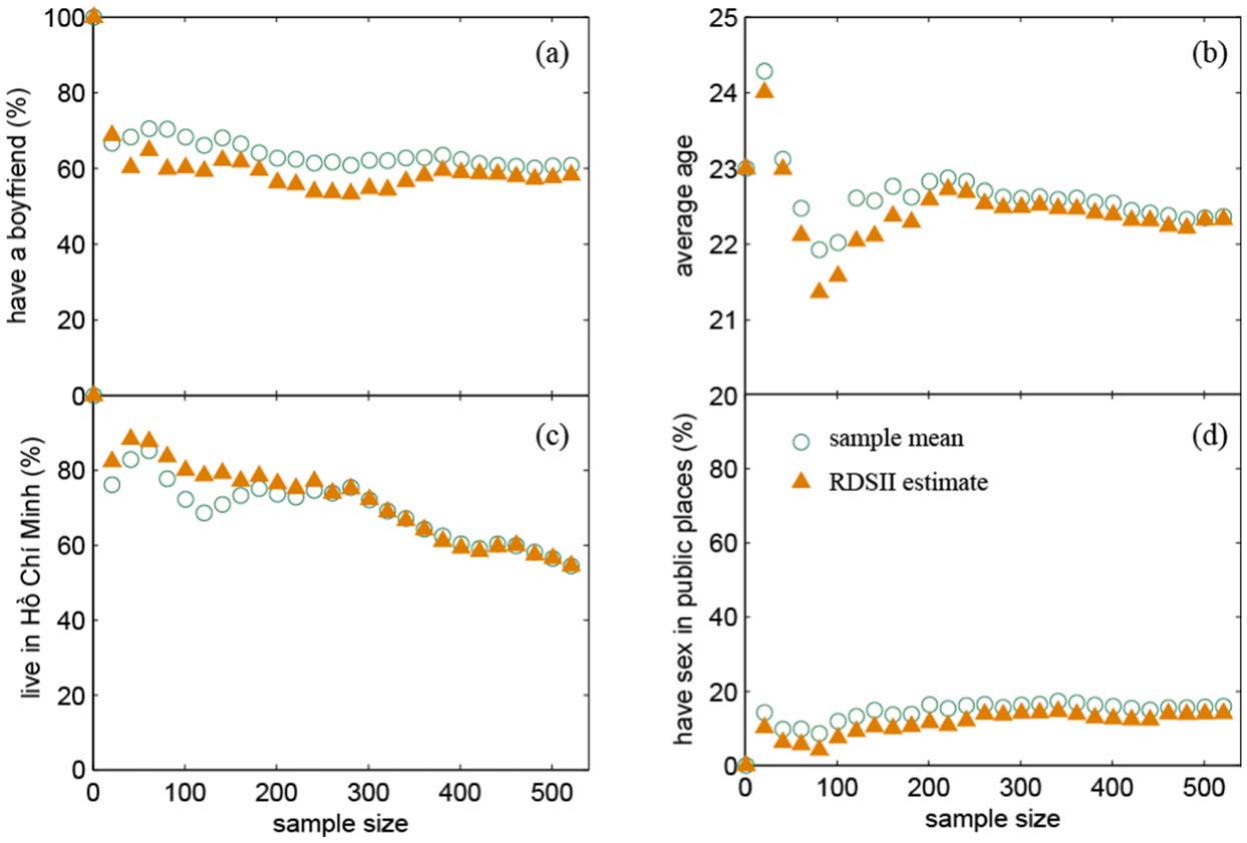
Change in sample composition with increasing sample size (not adjusted for network size): a) proportion that currently has a romantic relationship; b) average age; c) proportion living in Ho Chi Minh City (the only variable that did not stabilize); and d) proportion who had sex in a public place during the past six months. All variables available in the supplemental material.

Using the standard criteria in the literature [32], equilibrium was reached for all variables after a maximum of seven waves and a median of two waves. We also plotted the sample compositions with increasing sample sizes. Selected variables are shown in Fig. 3 and all variables are available in the supplementary material. Judging from these plots, the sample compositions stabilized well for all variables in the survey, with the exception of home province. The maximum absolute difference in RDSII estimated proportions comparing the full sample and the last 200 respondents among all the variables in the supplementary material, was 4.3% for estimates of proportions and 0.67 for estimated numeric values (sexual partner numbers, age and social network sizes).

### Characteristics of the Sample

**Figure 4:**
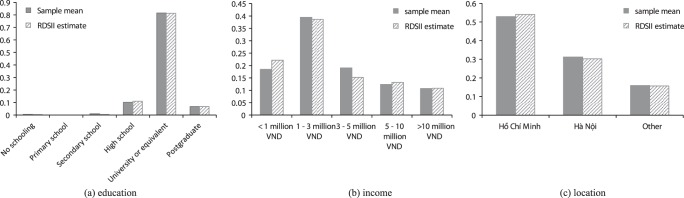
Sample proportions and estimated population proportions for a) education, b) income, and c) province.

The majority of the sample consisted of young persons with an estimated mean and median age of 22 years. The estimated proportion with education at vocational school, college or university was 87%. An estimated 67% used the Internet every day during the past month and an estimated 82% came from the two large metropolitan areas of Ho Chi Minh City and Hanoi (81% of the sample). The recruitment chains also penetrated outside the large metropolitan areas with 32 provinces represented out of 63 (Fig. 4a-c).

**Figure 5:**
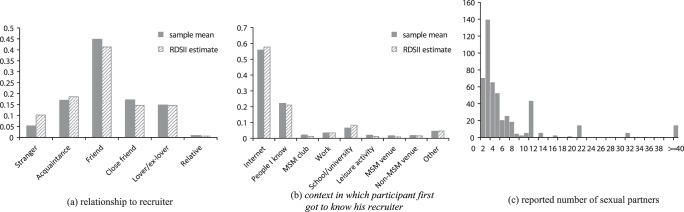
a) Relationship to recruiter, b) context in which participant first got to know his recruiter, and c) histogram of number of sex partners.

An estimated 98% (99% of the sample) preferred only men or preferred men to women as sexual partners, and 81% (81% of the sample) thought that same-sex marriage should be allowed in Vietnam. An estimated 92% (91% of the sample) had an existing relationship to their recruiter (an estimated 8% recruited by a stranger). Median number of sexual partners during the last six months was two (Fig. 5a-c).

## Discussion

We developed an automatic webRDS system to sample men who have sex with men (MSM) in Vietnam, a country in which same sex relationships are highly stigmatized and can lead to severe consequences if revealed to family members or colleagues [27]. We successfully used the system to sample and survey 676 MSM on a number of sensitive issues. We evaluated the independence of the seeds from the sample by showing that sample composition stabilized very well for all variables, possibly with the exception of home province. We used a varied set of incentives to stimulate participation and recruitment, which became rapid and robust, reaching 24 waves. The results indicate that webRDS could potentially be implemented at a low cost among Internet-using MSM as well as potentially becoming a valuable method for sampling other Internet-using populations.

Comparing national statistics and other published research data to our estimates show interesting similarities and dissimilarities that may reflect sampling bias, variability between data collection instruments and systematic differences between the sexually active Internet-using MSM population and the general population.

Using the RDSII estimator, 97% of the MSM population under study was estimated to be below 30 years of age and the sample mean and median ages were 22 years. By comparison, 43% of the adult male population in Vietnam is between 18 and 29 [33]. The lower mean age of sampled MSM compared to the national age distribution for men is consistent with an offline RDS study of MSM in Khanh Hoa, Vietnam, which reported a median ages of 24 years 34 and an RDS in Hanoi with median age of 2024 years 31. One online survey among visitors to Vietnamese MSM websites has been published and had a median age of 23 years with 18% stating an age above 30 years 31. Income distribution (Fig. 4b) is broadly consistent with the national average monthly per capita income for urban areas (2,130,000 VND, 2010 [35]). It is also comparable to data from the online survey among visitors to Vietnamese MSM websites [31] and the offline RDS in Hanoi 2008 [31], although inflation, economic growth and differential categorization of income levels precludes an exact comparison. An estimated 88% had some type of post-secondary education, including vocational training. This can be compared with 68% in the offline RDS in Hanoi 31 and 79% in the survey among visitors to Vietnamese MSM websites 31. The sample was heavily concentrated to the two large metropolitan areas of Ho Chi Minh City and Hanoi, with a population estimate of 84% for these cities combined. Ho Chi Minh City and Hanoi constitute approximately 55% of the urban population in Vietnam and about 16% of the national population 36,37. This is similar to the online banner survey on Vietnamese MSM websites where 74% came from Hanoi and HCMC 31. Explanation for the observed differences compared with national statistics may include migration of young MSM to the large cities, urban-rural differences in prevalence of male-male sex and different levels of access to the Internet. We did not find evidence that the mens social networks formed geographically isolated groups, which otherwise would have been a source of bias. The recruitment chains in our sample frequently crossed over between provinces. In total, 30% of all recruitment events took place between persons in different provinces. Additionally, like other social networks, MSM networks in Vietnam are most likely small-world networks 38, with short numbers of steps between provinces.

One percent stated that they preferred only women or preferred women to men as sexual partners. The banner survey on MSM sites [31] and an offline RDS in Hanoi with similar question [31] recorded 15% and 1.9% respectively for the same responses. A middle option (“Prefer women and men equally”) was available in these studies in contrast to our study, with 14% and 8% of answers respectively.

In summary, the webRDS reached a varied sample of largely young men concentrated in the two major cities of Vietnam, with an education higher than the average for the country. Whether these results indicate sampling bias or reflect differences between the general Vietnamese population and the Internet-using MSM population is difficult to assess.

There are limitations to this study. We excluded 13% (n = 85) of the submissions because of duplicated personal information or an age below 18 years. While this shows that the recruitment system did not work perfectly, it also shows the potential for eliminating duplicate submissions.

17.5% of completed surveys (115 surveys) included an IP number that had previously been registered in the system, which may signal duplicated submissions. However, it is important to note that IP-numbers are shared by all users at an Internet cafe and often by all users within a neighborhood. An array of non-Vietnamese IP-numbers is also used in Vietnam to access restricted sites like Facebook. We checked whether the final estimates were sensitive to exclusion of these submissions as well as of submission with very fast completion time, and did not find that this was the case. Similar protocols for quality check as those used in this study have been employed in other Internet-based surveys among MSM [39], [40]. Because sincere and insincere participants are likely to interact differently with web survey pages, analyzes of online behavioral data gathered during surveys may in the future provide a way to improve these protocols.

We opted for removal of ineligible participants after the study was concluded in order to observe recruitment behavior without outside involvement and to avoid running the risk of inadvertently stopping the survey. This procedure should not produce bias in the RDSII estimation if removal of ineligibles is made in a correct way. However, there will surely always remain questions as to the extent to which ineligibles have been fully removed or not. If future studies show that ineligibles can safely be removed without stopping global recruitment this is preferable.

We lack information as to the true participation rate as we do not have information on the proportion of invitation messages forwarded by the participants. However, we get some additional information by considering that on average, in order for recruitment to be sustained, each participant needs to recruit a minimum of one new participant. Persons who participated in the formative research for the study may be part of the sample, but it seems unlikely that this should have created important bias. The network size question did not exclude MSM living outside Vietnam and those of ages under 18 years. Potentially this may have underestimated e.g. the proportion of young persons. Eight percent of participants were recruited by a stranger. Other RDS studies among MSM have recorded similar proportions [41], [42], [43]. We do not think this caused serious bias in this study (see e.g [20]) but the issue should be monitored in future webRDS studies.

Although this study aimed to sample Internet-using MSM, access to the Internet, including literacy, will always be a limiting factor for representative sampling of MSM in general. Bio-markers will obviously also be challenging to collect with webRDS.

Although more than 600 submissions were received within five weeks, recruitment eventually died out despite being far from the total size of the Internet-using MSM population in Vietnam (at least 10,000 persons). One explanation may be local as opposed to global saturation. It is very common in acquaintance networks that an individual’s neighbors are connected with each other. The risk that people will try to recruit acquaintances who have already been recruited will therefore increase over time, decreasing the effective reproductive number and could result in a curve shape similar to the one in Fig. 2 [44].

WebRDS may have several advantages over standard offline RDS and other Internet-based sampling methods for hidden groups. In comparison to standard RDS it may allow for representative sampling of hidden groups without geographical limits and can potentially generate larger samples than standard RDS. This would also enable valuable data on variables for which design effects are high [14]. Online networks may also cross social boundaries more often than offline social networks (decreased homophily) and are likely to generally produce larger average personal network sizes than offline networks, both of which can decrease design effects [15]. Individuals who for various reasons prefer not to access an RDS survey office physically may additionally be willing to take part in an anonymous web survey.

Web-RDS will in most cases entail a lower costs than a standard RDS study. Cost for monetary incentives were in our study on average 5.9 USD per participant in the cleaned sample (3353 USD in total). Staff hours to interact with seeds, deliver incentives, monitoring invalid submissions etc, totaled one month full-time equivalents (FTE). Adjustment of the site to appeal to the local target group is technically easy but requires formative research. For comparison, an offline RDS would have shared similar costs for incentives and formative research about the study population (see e.g. [45]) but would also require a survey office and at least five months staffing (conservative FTE estimate). We are currently improving the ease of use of the software and researchers interested in the survey system are welcome to contact the authors.

Current online recruitment of hidden groups is based on self-selected samples of persons who access certain Internet sites and click banner ads for a study. These surveys often have participation rates of a few in a thousand to a few in a hundred [23], [24]. As compared with such online sampling, successful webRDS is likely to achieve considerably reduced self-selection bias, because sustained recruitment is likely to be highly correlated to high participations rates.

In summary, we developed a webRDS system to sample men who have sex with men in Vietnam and showed that is was possible to survey participants on a range of sensitive issues, including sexual behavior, while sustaining recruitment and achieving equilibrium. The results indicate that the method could potentially be implemented at low cost among Internet-using MSM. With further evaluation and among suitable population groups, Internet-based RDS could become a promising method for representative sampling online.

## Supporting Information

Figure S1 **Sample proportions and RDSII estimates with increased sample size.** Cleaned sample used. The curves for sample groups with other criteria are similar.
(EPS)

Table S1 **RDSII estimates for samples with progressively stricter inclusion criteria.**
(DOCX)
