# Supplementary material for: Implementation of Web-Based Respondent-Driven Sampling among Men Who Have Sex with Men in Vietnam
Source: PLoS One. 2012 Nov 12;7(11):e49417. doi: 10.1371/journal.pone.0049417 (PMC3495883; doi:10.1371/journal.pone.0049417)
Supplement: Table S1 — RDSII estimates for samples with progressively stricter inclusion criteria. (DOCX) [file pone.0049417.s002.docx]

Implementation of Web-Based Respondent-Driven Sampling among Men who Have Sex with Men in Vietnam

*Supporting Information*

1. **RDS estimates for sample data with progressively stricter criteria**

We checked all surveys for other signs of duplication or invalid submission. We flagged surveys containing a repeated IP number, deviating answers, or short completion times. We analyzed the sensitivity of the estimates to inclusion and exclusion of these flagged submissions. Specifically, we compared the RDSII estimates generated from the full sample of non-seed submissions with valid age with the RDSII estimates generated from groups with progressively stricter inclusion criteria according to the following:

**Non-Strict:**

All non-seed respondents with valid age (≥18);

**Cleaned sample^[[1]](#footnote-1)^:**

All non-seed respondents with valid age (≥18);

Exclude submissions with repeated email, Yahoo! Chat ID or telephone number;

**Strict:**

All non-seed respondents with valid age (≥18);

Exclude submissions with repeated email, Yahoo! Chat ID, telephone number;

Exclude submissions with repeated IP address;

**Very strict:**

All non-seed respondents with valid age (≥18);

Exclude submissions with repeated email, Yahoo! Chat ID, telephone number;

Exclude submissions with repeated IP address;

Exclude submissions with short completion times (< 3 minutes), submissions stating no education (rare in Vietnam), or submission stating six-month partner numbers of above 1,000.

RDSII estimates of all 17 questions for the above sample groups with progressively stricter inclusion criteria are listed in Table 1. The differences between groups are small. The average absolute differences in proportional estimates when comparing the full sample (non-strict criteria) to the other groups, is less than 0.64% (maximum difference 6.6%), and the average absolute differences in numeric estimates is 0.12 (maximum difference 0.30), see Table 1.

| **Variable** | **Non-Strict (n=634)** | **Cleaned sample (n=571)** | **Strict (n=490)** | **Very**  **Strict**  **(446)** |
| --- | --- | --- | --- | --- |
| ***Proportional estimates (%)*** |  |  |  |  |
| *1. Have a boyfriend now* | 57.5 | 56.6 | 58.0 | 57.4 |
| *2. Longest relationship with men ≥ 6 months* | 52.0 | 52.5 | 51.4 | 51.3 |
| *3. Prefer “good looking” when looking for someone for sex* | 47.6 | 48.5 | 49.4 | 48.6 |
| *4. Prefer “faithful” when looking for someone for long-term relationship* | 39.2 | 41.1 | 41.5 | 39.5 |
| *5. Support same sex marriage* | 77.3 | 79.0 | 80.0 | 81.2 |
| *6. Prefer only men as sexual partners* | 68.0 | 68.3 | 67.3 | 66.2 |
| *7. Had sex in public places during past 6 months* | 14.2 | 13.4 | 11.8 | 12.2 |
| *8. Have some education after high school (vocational training, college or university)* | 87.6 | 88.1 | 87.2 | 86.5 |
| *9. Monthly income ≥ 5 million VND* | 24.0 | 24.0 | 25.7 | 25.9 |
| *10. Live in Hồ Chí Minh city* | 53.4 | 54.1 | 55.3 | 55.9 |
| *11. Use the Internet everyday* | 57.0 | 59.6 | 63.6 | 62.2 |
| *12. Recruited by friend* | 40.9 | 41.3 | 42.9 | 42.3 |
| *13.First go to know recruiter through friends, lovers or relatives* | 21.4 | 21.1 | 20.0 | 20.3 |
| ***Numeric estimates*** |  |  |  |  |
| *14. Number of men had sex with during past 6 months* | 4.03 | 4.07 | 3.96 | 4.05 |
| *15. Average age* | 22.23 | 22.22 | 22.07 | 22.06 |
| *16. Number of MSM friends* | 6.74 | 6.76 | 6.91 | 7.04 |
| *17. Number of MSM friends who use Internet* | 5.23 | 5.24 | 5.43 | 5.47 |

^*^For categorical questions one answer per question is shown

1. **Equilibrium curves for all variables surveyed in study**

To get an overview of whether the sample reached equilibrium, we plot both the sample proportion and the RDSII estimates along with the increased sample size for all variables surveyed in this study (see Figure 1).

To measure the change during the last part of the sampling process when the sampling compositions should have stabilized, we calculate the changes in the sample compositions comparing the full sample and the full sample excluding the last 200^th^ respondents:

Or

$$\Delta\hat{y}^{RDSII}=\hat{y}_{n}^{RDSII}-\hat{y}_{n-200}^{RDSII}$$

We can see that except for answers for province of residence, the sample proportion and RDS estimates for all other variables became quite stable after 100~200 submissions, the maximum absolute difference between last 200 respondents, is 5.3% for raw sample proportions, 4.3% for RDSII estimated proportions, and 0.67 for the RDSII estimated average numerical variables.

The decreased proportions of respondents from Hồ Chí Minh City, indicates that the recruitment chains on average spread from Hồ Chí Minh City to other provinces. The stable estimates for variables not related to place of residence suggest that there is little difference between provinces regarding the social and sexual behaviors/opinions studied here.

Four seeds were selected from Hoa Binh and as can be expected from the RDS Markov process that should create independence between the seeds and the final sample, the proportion from the small province of Hoa Binh was only an estimated 1.6%.

There may be a number of possible reasons for not reaching stable proportions for place of residence in the present study sample. Internet-using persons from countryside may have less access to computers in the home and thus take longer time before completing surveys. The network may also be less developed between the large cities and the countryside and social networks between any two rural provinces may be less developed than between rural and urban provinces causing recruitments between rural provinces to go through urban hubs.

1. This is the cleaned sample discussed in the paper. [↑](#footnote-ref-1)
